# Overexpression of *SiLEA_2-6* from *Saussurea involucrata* Enhances Salt and Osmotic Tolerance in *Arabidopsis thaliana*

**DOI:** 10.3390/plants15203154

**Published:** 2026-10-15

**Authors:** Saisai Wang, Xiaoyan Wu, Yifei Chen, Weixiang Zhou, Zhenhai Zhang, Hongxiang Zhang, Bo Cui, Ruina Liu, Wenwen Xia, Jin Li, Jianbo Zhu

**Affiliations:** College of Life Science, Shihezi University, Shihezi 832000, China; wssshzu@163.com (S.W.); wxy1245891567@163.com (X.W.); cyfshz@126.com (Y.C.); zwx2000301@163.com (W.Z.); 18690918176@163.com (Z.Z.); 18134856295@163.com (H.Z.); cuibo231224@163.com (B.C.); lrnshz@126.com (R.L.); xiashz@126.com (W.X.)

**Keywords:** *Saussurea involucrata*, *SiLEA_2-6*, salt tolerance, osmotic stress, subcellular enrichment

## Abstract

LEA_2 proteins are intrinsically disordered proteins implicated in plant abiotic stress tolerance; however, their functional characterization under salt and osmotic stress, particularly regarding stress-inducible peripheral redistribution, remains limited. SiLEA_2-6, an LEA_2 protein from the extreme-cold-tolerant species *Saussurea involucrata*, was identified from a cold-stress cDNA library, but its role in salt and osmotic tolerance remains unknown. In the present study, transgenic *Arabidopsis thaliana* Col-0 lines constitutively overexpressing *SiLEA_2-6*–eGFP were generated. Overexpression significantly enhanced salt and osmotic tolerance across germination, vegetative, and reproductive stages, as evidenced by improved germination rates, root elongation, survival, and physiological performance. Physiological analyses revealed improved membrane integrity (REL and MDA accumulation), enhanced osmotic adjustment, and preserved chlorophyll content. Subcellularly, SiLEA_2-6–eGFP displayed diffuse cytoplasmic distribution with intracellular punctate structures under non-stress conditions; within 60 min of salt or osmotic stress, it showed pronounced peripheral association, notably at guard cells and stomatal-adjacent regions. These findings demonstrate that SiLEA_2-6 enhances salt and osmotic tolerance in *Arabidopsis*, with stress-inducible peripheral enrichment consistent with a potential membrane-associated protective function. This study adds to the growing evidence that LEA_2 proteins confer tolerance not only to cold/freezing but also to salt and osmotic stress.

## 1. Introduction

Soil salinity and drought are among the most severe abiotic stresses that limit global crop productivity and affect over 800 million hectares of arable land worldwide [1]. Both stresses induce cellular dehydration, leading to osmotic imbalance, ion toxicity, oxidative damage, and ultimately membrane destabilization and photosynthetic impairment [2,3]. To overcome these challenges, plants have evolved complex adaptive mechanisms, including the accumulation of stress-responsive proteins that protect cellular structures and maintain homeostasis [4]. Among these, late embryogenesis abundant (LEA) proteins have emerged as key players in plant abiotic stress tolerance [4,5].

LEA proteins were first identified as seed-specific proteins that accumulate during the late maturation stage of embryogenesis, protecting desiccating seeds from cellular damage [6]. They are now known to be expressed in vegetative tissues under various stress conditions, including salinity, drought, and cold [4]. LEA proteins belong to the broader class of intrinsically disordered proteins (IDPs), characterized by flexible conformations that enable rapid structural transitions and interactions with multiple cellular targets [7]. This structural plasticity underlies their diverse protective functions, including membrane stabilization, ROS scavenging, chaperone-like activity, and prevention of protein aggregation during dehydration [8,9,10].

The LEA superfamily is conventionally divided into eight subfamilies based on conserved sequence motifs [11]. Among the LEA subgroups, LEA_2 (Pfam: PF03168) is noteworthy for its relatively atypical properties. In contrast to classical LEA proteins, which are highly hydrophilic, LEA_2 members tend to be more hydrophobic, representing a distinct evolutionary branch within the superfamily [5]. Despite this structural divergence, multiple studies have demonstrated that LEA_2 proteins confer robust drought and salt stress tolerance in plants. For instance, Zheng et al. [5] isolated *IpLEA*, a LEA_2 group gene from the halophyte *Ipomoea pes-caprae*, and demonstrated that it is widely expressed across different organs and strongly induced by salt, osmotic, oxidative, freezing, and ABA treatments. Similarly, *SmLEA2* from *Salvia miltiorrhiza*, which phylogenetically belongs to the LEA_2 subfamily, improves the growth performance of both *Escherichia coli* and transgenic *S. miltiorrhiza* under drought and salt conditions, with overexpressing lines exhibiting significantly increased superoxide dismutase (SOD) activity and reduced lipid peroxidation [12]. *GmLEA2-1* from soybean shares low sequence similarity with other LEA_2 members across species but retains conserved protective functions, and its overexpression in transgenic *Arabidopsis* confers enhanced tolerance to drought and salt stress, as evidenced by greater fresh weight, root length, and lateral root density [13]. *CkLEA2-3* from the desert legume *Caragana korshinskii* likewise enhances tolerance to ABA, osmotic, and salt stress during seed germination and confers drought resistance during seedling development when overexpressed in *Arabidopsis* [14].

Although LEA_2 proteins have been implicated in cold and freezing tolerance in several species [15,16,17], their dynamic subcellular behavior under salt and osmotic stress remains largely unexamined. *Saussurea involucrata* (*S. involucrata*), a perennial herb native to the high-altitude regions of Central Asia, is renowned for its extreme tolerance to multiple abiotic stresses, including freezing, drought, and intense ultraviolet (UV) radiation [18]. This remarkable resilience makes the snow lotus a valuable resource for cloning stress-resistant genes. Several LEA genes from this species have already been shown to enhance stress tolerance when heterologously expressed. For example, *SiDHN* overexpression in tomato and tobacco reduces drought-induced membrane damage and enhances ROS scavenging [19,20], and SiLEA4 improves both *Escherichia coli* viability under cold stress and transgenic tomato yield and freezing tolerance [21].

*SiLEA_2-6* (unpublished data; GenBank accession PZ794267, release 1 December 2026) is a 924-bp CDS encoding a 307-amino-acid protein belonging to the LEA_2 subfamily (Pfam PF03168). The conserved domain architecture of this gene is closely related to that of *Arabidopsis* AtLEA14, a member of the LEA_2 family, which is implicated in stress-responsive processes. The gene was originally isolated from a cold-stress cDNA library of *S. involucrate* [22]. Given the stress-adapted genetic background of *S. involucrata* and the demonstrated role of LEA_2 proteins in abiotic stress protection, SiLEA_2-6 represents a promising candidate for conferring salt and osmotic tolerance in heterologous systems. Moreover, as LEA_2 proteins are proposed to interact with membrane systems [10,23], the subcellular distribution of SiLEA_2-6 under stress warrants investigation.

To test these possibilities, we generated transgenic *Arabidopsis* (Columbia-0, Col-0) lines constitutively overexpressing a *SiLEA_2-6*–eGFP fusion construct and evaluated their performance under salt and osmotic stress across seed germination, vegetative growth, and the reproductive phase. We hypothesized that *SiLEA_2-6* overexpression would enhance salt and osmotic tolerance across developmental stages and that the fusion protein would exhibit stress-inducible redistribution within the cells.

## 2. Result

### 2.1. Construction and Molecular Verification of the SiLEA_2-6 Overexpression Vector

**Figure 1:**
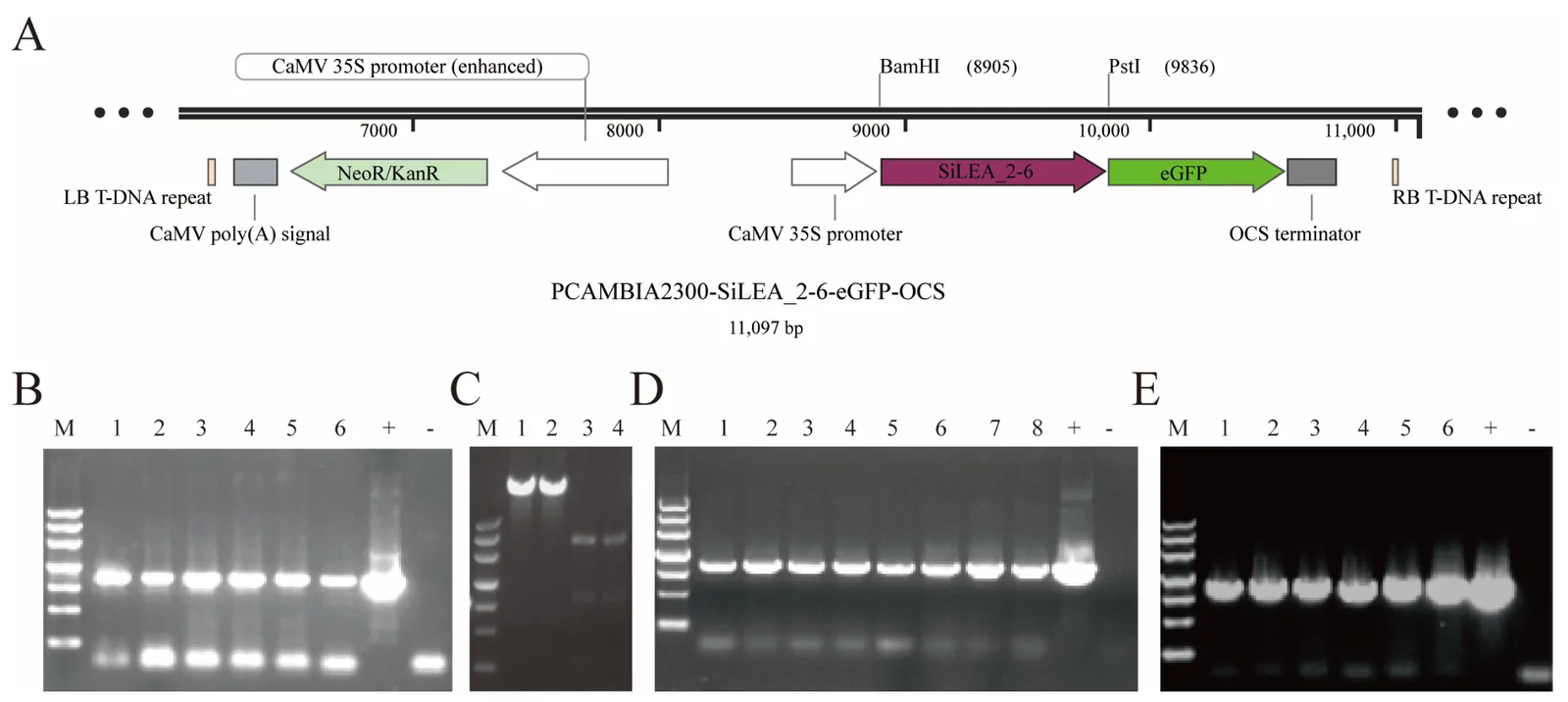
Construction and stepwise verification of the SiLEA_2-6 overexpression vector. (**A**) Schematic map of pCAMBIA2300-*SiLEA_2-6*-eGFP-OCS (11,097 bp). The T-DNA region contained an enhanced CaMV 35S promoter, *SiLEA_2-6* CDS with the stop codon removed (to allow in-frame fusion with eGFP), eGFP, and the OCS terminator. The NptII/kanamycin-resistance cassette and CaMV poly(A) signal were located outside the expression cassette. LB and RB denote the left and right T-DNA borders, respectively. The *BamH* I and *Pst* I cloning sites used for insertion are indicated. (**B**) Colony PCR of *SiLEA_2-6* cloned into the pMD19-T vector. M, DNA marker III (Takara, Dalian, China); fragment sizes from top to bottom: 4500, 3000, 2000, 1200, 800, 500, 200 bp; lanes 1–6, individual colonies; “+”, positive control; “−”, negative control. The expected amplicon size was ~921 bp. (**C**) *BamH* I and *Pst* I double digestion. Lanes 1–2, empty pCAMBIA2300-eGFP vector (no insert, ~9.0 kb linearized backbone); lanes 3–4, pMD19-T–*SiLEA_2-6* intermediate cloning vector, showing the ~921 bp SiLEA_2-6 insert (lower band) and the linearized vector backbone (~2.7 kb, upper band). M, DNA marker III. (**D**) Colony PCR of pCAMBIA2300-*SiLEA_2-6*-eGFP recombinant clones in *Escherichia coli*. M, DNA marker III; lanes 1–6, individual colonies; “−”, negative control; “+”, positive control. (**E**) Colony PCR of *Agrobacterium tumefaciens* GV3101 harboring the recombinant vector. M, DNA marker III; lanes 1–6, individual colonies; “+”, positive control; “−”, negative control.

Based on the *SiLEA_2-6* mRNA sequence originally cloned from *S. involucrata* cDNA, the coding region was 924 bp. In this study, the known CDS was used as the starting material, with its native stop codon removed, to construct an *Arabidopsis* overexpression vector (Figure 1A). The CDS fragment was first cloned into the pMD19-T intermediate vector, positive clones were obtained by ampicillin selection, and verified by colony PCR (Figure 1B) and Sanger sequencing. The insert was then released by *BamH* I and *Pst* I double digestion, and agarose gel electrophoresis confirmed the expected fragment size of ~921 bp (Figure 1C). The recovered fragment was directionally subcloned into pCAMBIA2300-eGFP, and the recombinants were selected using kanamycin and verified by colony PCR (Figure 1D). Finally, the recombinant overexpression construct was introduced into *Agrobacterium tumefaciens* GV3101, and colony PCR confirmed the successful delivery of the construct into agrobacteria (Figure 1E), enabling the generation of transgenic *Arabidopsis* lines for phenotypic analysis.

### 2.2. Generation and Molecular Characterization of Transgenic Arabidopsis Lines

**Figure 2:**
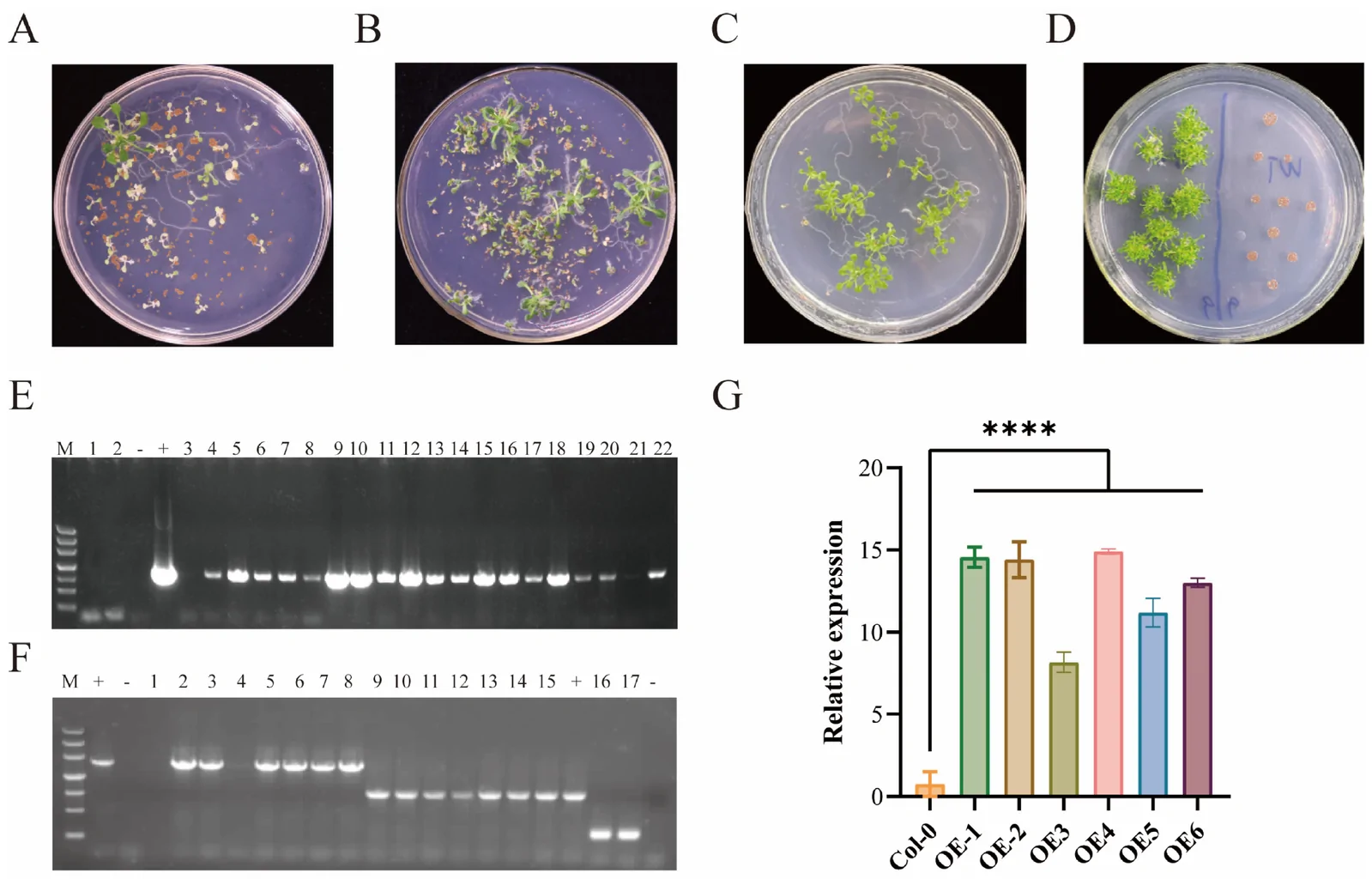
Kanamycin resistance screening and molecular verification of transgenic *Arabidopsis thaliana* lines. (**A**–**D**) Kanamycin resistance screening of seeds on the selection medium. (**A**) T_1_ generation; (**B**) T_2_ generation; (**C**) T_3_ generation; (**D**) T_3_ generation alongside wild-type Col-0 (right panel). Green seedlings indicated resistant individuals. (**E**) Genomic DNA-level PCR detection of *SiLEA_2-6* in the transgenic lines. M, DNA marker III; lanes 1–2, Col-0 (wild type); lanes 3–22, kanamycin-resistant lines; “+”, positive control; “−”, negative control. (**F**) Reverse transcription PCR (RT-PCR) detection. Total RNA was reverse-transcribed into cDNA, and PCR products were separated by 1.0% agarose gel electrophoresis. Lanes 1 and 16–17, Col-0 controls; lanes 2–8, *SiLEA_2-6*-eGFP fusion transcript; lanes 9–15, *SiLEA_2-6* transcript; “+”, positive control; “−”, negative control. (**G**) Relative expression levels of *SiLEA_2-6* in wild-type (Col-0) and transgenic overexpression lines (OE1–OE6). Statistical significance was determined using one-way ANOVA with Turkey’s multiple comparison test. ns, not significant; ****, *p* < 0.0001.

Transgenic *Arabidopsis* (Col-0 background) was generated using *Agrobacterium*-mediated floral dip transformation. Kanamycin selection on 1/2 Murashige and Skoog (MS) solid medium identified putative transgenic T_1_ *Arabidopsis* plants, which remained green and continued to develop, whereas non-transgenic seedlings were bleached and failed to develop (Figure 2A). Positive T_1_ individuals were advanced, and the progeny were screened sequentially through the T_2_ and T_3_ generations, with resistant green seedlings recovered in each generation (Figure 2B,C). By the T_3_ generation, resistant lines were distinguished from the wild-type Col-0 control, which showed no resistance and was arrested as bleached seedlings under the same selection (Figure 2D).

Genomic PCR confirmed the integration of *SiLEA_2-6* in the resistant lines (lanes 3–22), whereas the Col-0 control (lanes 1–2) and negative control were negative (Figure 2E). Transgene transcription was verified by reverse transcription PCR (RT-PCR) followed by agarose gel electrophoresis. The *SiLEA_2-6*-eGFP fusion transcript was detected in lanes 2–8, and *SiLEA_2-6* transcripts were detected in lanes 9–15, with no amplification in Col-0 controls (lane 1 and lanes 16–17) (Figure 2F). These results verified the stable genomic integration and expression of *SiLEA_2-6* in the transgenic lines, enabling subsequent phenotypic analysis.

Among 7 independent T_3_ lines obtained from distinct transformation events, two lines (OE1, lane 2/9; and OE2, lane 6/13) were selected for detailed phenotypic and physiological analysis based on robust SiLEA_2-6-eGFP expression confirmed by RT-PCR (Figure 2F) and qRT-PCR (Figure 2G). qRT-PCR quantification demonstrated significantly higher transcript levels in both OE lines compared to WT, validating successful overexpression as the foundation for all subsequent experiments.

### 2.3. Overexpression of SiLEA_2-6 Enhances Seed Germination and Root Growth Under Salt and Osmotic Stress

**Figure 3:**
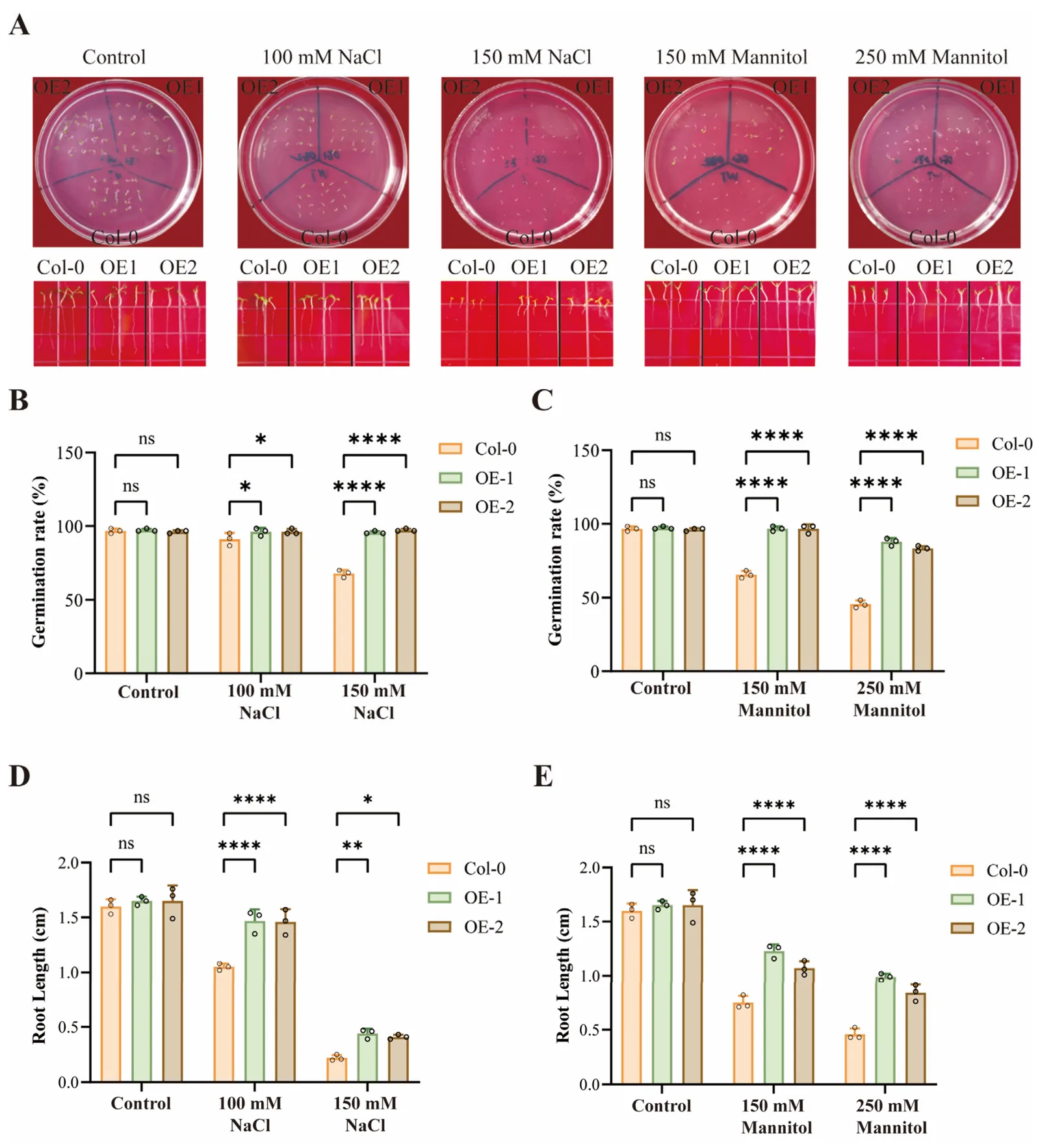
*SiLEA_2-6* overexpression enhances seed germination and root growth under salt and osmotic stress conditions in *Arabidopsis*. (**A**) Representative images of 7-day-old seedlings and germinated seeds grown on control, NaCl-supplemented (100 and 150 mM), or mannitol-supplemented (150 and 250 mM) MS medium. Scale bar = 1 cm. (**B**,**C**) Germination rates under salt (**B**) and mannitol (**C**) stress. (**D**,**E**) Primary root length under salt (**D**) and mannitol (**E**) stress conditions. Data are presented as mean ± SD (*n* = 3 biological replicates). Statistical significance was determined using two-way ANOVA with Dunnett’s post-hoc test. ns, not significant; *, *p* < 0.05; **, *p* < 0.01; ****, *p* < 0.0001.

To assess the functional role of *SiLEA_2-6*, transgenic *Arabidopsis* lines (OE1 and OE2; selected from 7 T_3_ lines as described in Section 2.2) and wild-type (Col-0) were subjected to salt (NaCl) and osmotic (mannitol) stresses. Under non-stress conditions (control), all genotypes showed near-100% germination and comparable primary root lengths (approximately 1.65 cm), with no statistically significant differences among WT, OE1, and OE2 (Figure 3A–E).

Under salt stress, OE1 and OE2 maintained significantly higher germination rates than that of WT. At 100 mM NaCl, the difference was significant, and at 150 mM NaCl, WT germination was markedly reduced, whereas that of both OE lines remained high (Figure 3B). Root growth was also better preserved in the transgenic lines; at 100 mM NaCl, OE roots were approximately 0.4 cm longer than WT, and at 150 mM NaCl, WT root length was approximately half that of OE1/OE2 (Figure 3D).

Similarly, OE1 and OE2 exhibited significantly improved germination under osmotic stress imposed by mannitol compared to WT at both 150 mM and 250 mM (Figure 3C). Primary root elongation was less inhibited in the transgenic lines: at 150 mM mannitol, OE1 and OE2 roots were approximately 0.48 cm and 0.32 cm longer than WT, respectively, and at 250 mM mannitol, the differences increased to approximately 0.53 cm and 0.38 cm, respectively (Figure 3E).

Collectively, these results demonstrate that *SiLEA_2-6* overexpression enhances tolerance to salt and osmotic stress by promoting seed germination and sustaining root growth under adverse conditions.

### 2.4. SiLEA_2-6 Overexpression Improves Physiological Tolerance to Drought and Salt Stress at the Vegetative Stage

**Figure 4:**
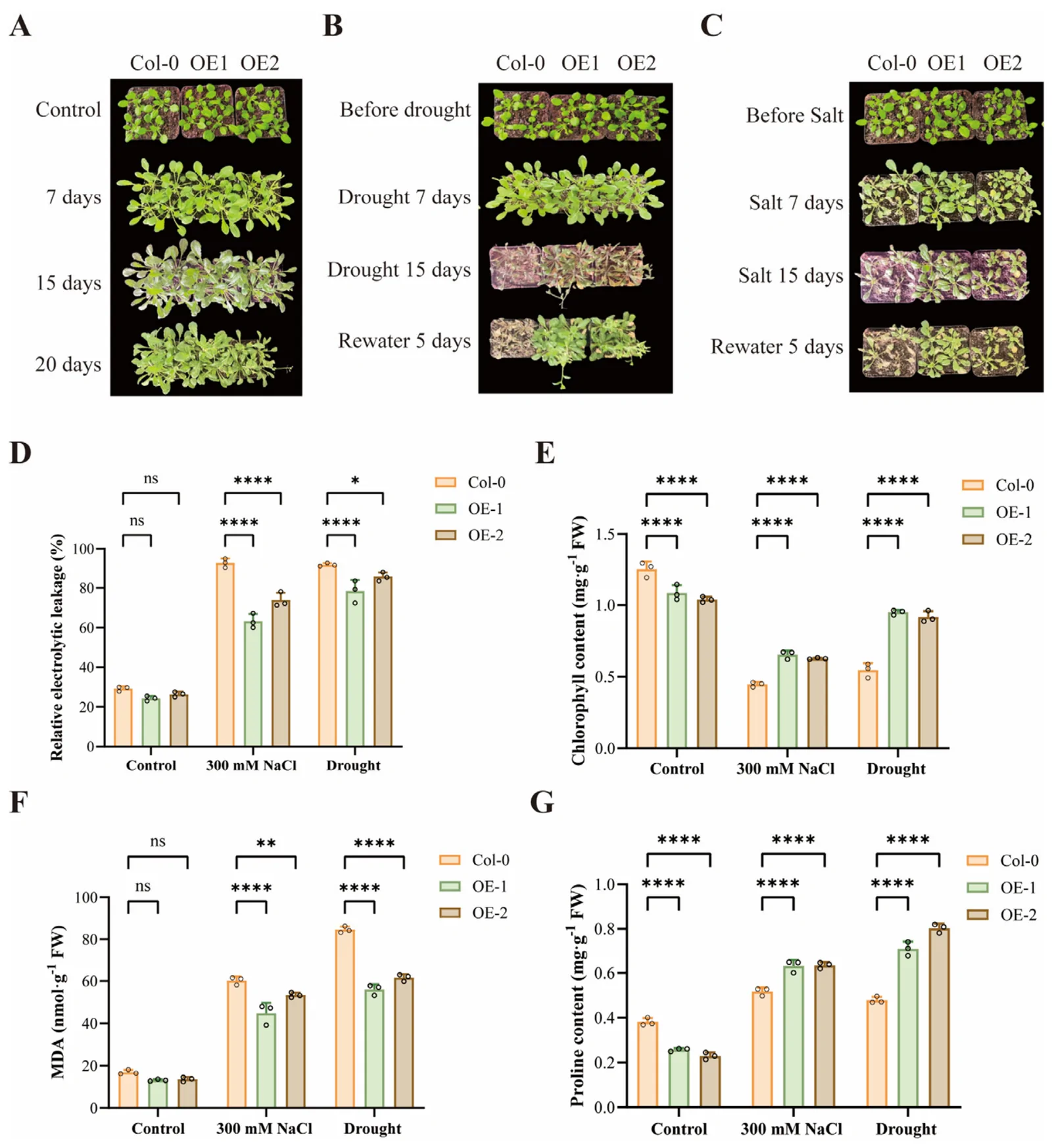
SiLEA_2-6-overexpressing *Arabidopsis* plants exhibited enhanced drought and salt tolerance at the vegetative stage. (**A**) Phenotypic comparison under well-watered, nonstress control conditions (photographed at the same vegetative stage as that used for subsequent stress treatments). (**B**) Phenotypic comparison under drought stress conditions. Plants were photographed before treatment, after 7 and 15 d of withholding water, and after 5 d of rewatering. (**C**) Phenotypic comparison under salt stress (300 mM NaCl). Plants were photographed before treatment, after 7 and 15 d of salt treatment, and after 5 d of rewatering treatment. (**D**–**G**) Physiological parameters under control, drought (withholding water for 15 days), and salt stress (300 mM NaCl for 15 days) conditions. (**D**) Relative electrolyte leakage (%); (**E**) chlorophyll content (mg·g^−1^·FW); (F) MDA content (nmol∙g^−1^∙FW); (**G**) proline content (mg·g^−1^ FW). Data are presented as mean ± SD (*n* = 3). Statistical significance was determined using two-way ANOVA with Dunnett’s post-hoc test. ns, not significant; *, *p* < 0.05; **, *p* < 0.01; ****, *p* < 0.0001.

Under well-watered, non-stress conditions, OE1, OE2, and WT (Col-0) plants at the vegetative stage maintained comparable growth over time (7, 15, and 20 days; Figure 4A), confirming that the observed phenotypic differences under stress were attributable to the treatments rather than developmental timing. The basal physiological parameters were also similar, with no significant differences in relative electrolyte leakage (REL), chlorophyll content, malondialdehyde (MDA) levels, or proline accumulation (Figure 4D–G).

Under drought stress (withholding water), phenotypic divergence became evident over time. After 7 days without irrigation, the WT and transgenic lines appeared similar; however, by day 15, the WT plants exhibited severe wilting, leaf senescence, defoliation, and chlorosis, whereas OE1 and OE2 maintained better turgor and foliar greenness than the WT. After 5 d of rewatering, transgenic plants recovered markedly, resuming growth with substantial refoliation and bolting, whereas WT plants showed limited recovery (Figure 4A). Consistent with these phenotypes, drought-stressed OE lines displayed significantly lower REL and MDA content, together with higher chlorophyll retention, indicating preserved membrane integrity and attenuated lipid peroxidation (Figure 4D–F). Proline accumulation was also significantly elevated in OE1 and OE2 plants under drought conditions, reflecting enhanced osmotic adjustment (Figure 4G).

Under salt stress (300 mM NaCl), WT plants developed pronounced wilting, chlorosis, and necrosis after 7 and 15 d, with mortality evident by day 15. In contrast, OE1 and OE2 showed markedly reduced symptom severity and retained greater green biomass, and upon rewatering, transgenic plants exhibited superior regreening and survival relative to WT (Figure 4C). Physiologically, OE lines maintained higher chlorophyll content and lower electrolyte leakage and MDA levels under salt treatment, alongside significantly increased proline accumulation (Figure 4D–G).

Collectively, these results demonstrate that *SiLEA_2-6* overexpression enhances drought and salt tolerance during the vegetative stage by safeguarding cell membrane integrity, sustaining photosynthetic pigment levels, limiting oxidative damage, and promoting osmotic adjustment and post-stress recovery.

### 2.5. SiLEA_2-6 Overexpression Maintains Chlorophyll Retention in Leaf Discs Under Salt and Osmotic Stress

**Figure 5:**
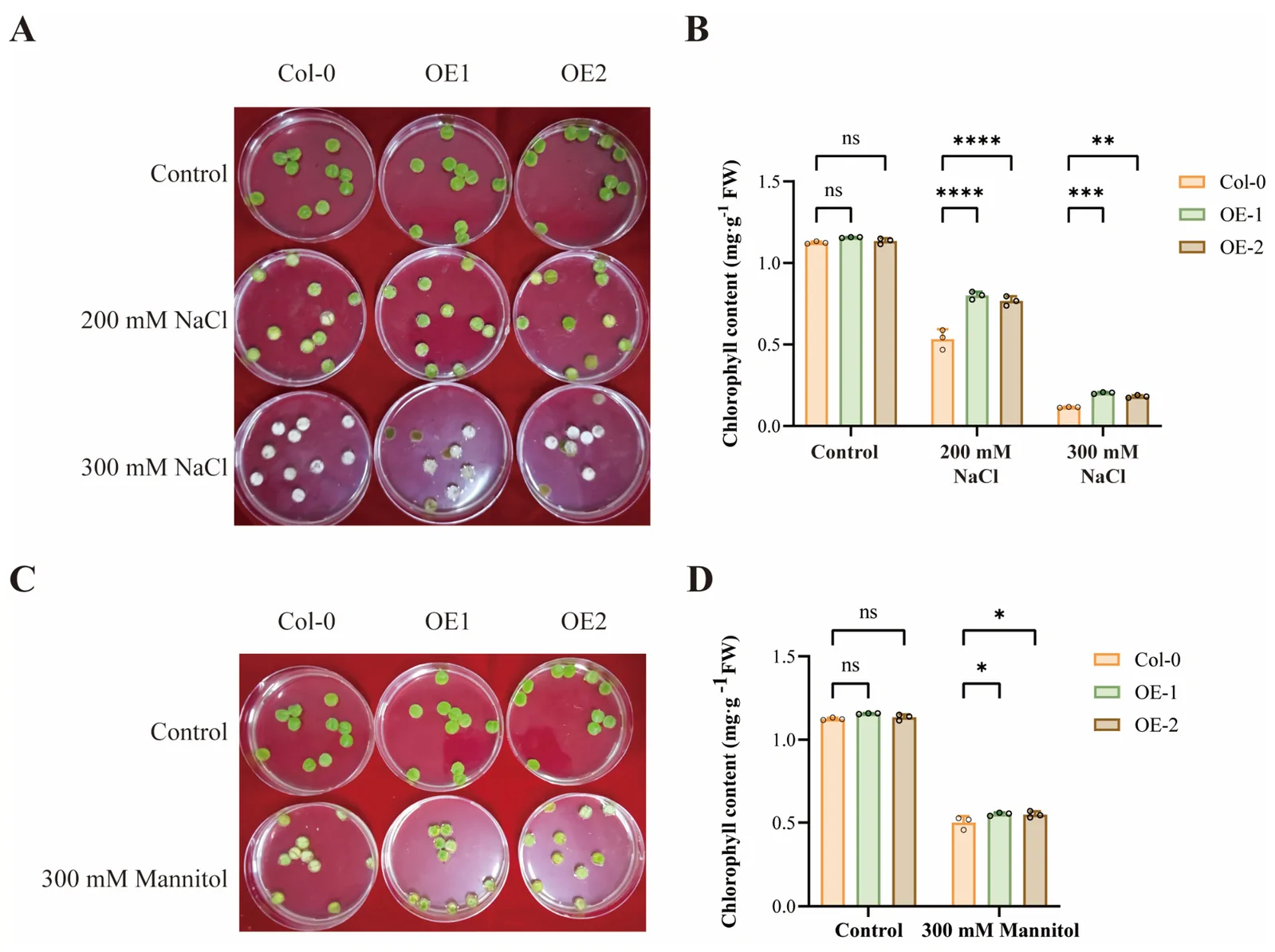
Detached leaf discs from three-week-old SiLEA_2-6-overexpressing *Arabidopsis* maintained chlorophyll levels under salt and osmotic stress. (**A**) Appearance of leaf discs after incubation under control, 200 mM NaCl, and 300 mM NaCl conditions. (**B**) Chlorophyll content (mg·g^−1^ FW) under NaCl treatments. (**C**) Appearance of leaf discs after incubation under control and 300 mM mannitol conditions. (**D**) Chlorophyll content (mg·g^−1^·FW) under mannitol treatment. Values are expressed as mean ± SD (*n* = 3). Statistical significance was determined using two-way ANOVA with Dunnett’s post-hoc test. ns, not significant; *, *p* < 0.05; **, *p* < 0.01; ***, *p* < 0.001; ****, *p* < 0.0001.

To further evaluate stress tolerance at the vegetative stage, detached leaf discs from three-week-old WT (Col-0), OE1, and OE2 plants were incubated under control conditions or exposed to NaCl (200 or 300 mM) or mannitol (300 mM). Under non-stress conditions, all genotypes showed similar green coloration and chlorophyll content. At 200 mM NaCl, WT discs exhibited significantly greater bleaching than OE1 and OE2, with correspondingly lower chlorophyll content. At 300 mM NaCl, WT discs were nearly completely bleached, whereas transgenic discs retained substantially more green tissue and significantly higher chlorophyll levels (Figure 5A,B). Similarly, under 300 mM mannitol, WT discs displayed more severe whitening and lower chlorophyll content than OE lines (Figure 5C,D). These results demonstrate that *SiLEA_2-6* overexpression preserves photosynthetic tissues from salt- and osmotic-induced chlorophyll degradation, consistent with the whole-plant drought and salt tolerance observed at the vegetative stage.

### 2.6. SiLEA_2-6 Overexpression Attenuates Salt- and Osmotic-Stress-Induced Chlorophyll Loss and Lipid Peroxidation at the Reproductive Stage

**Figure 6:**
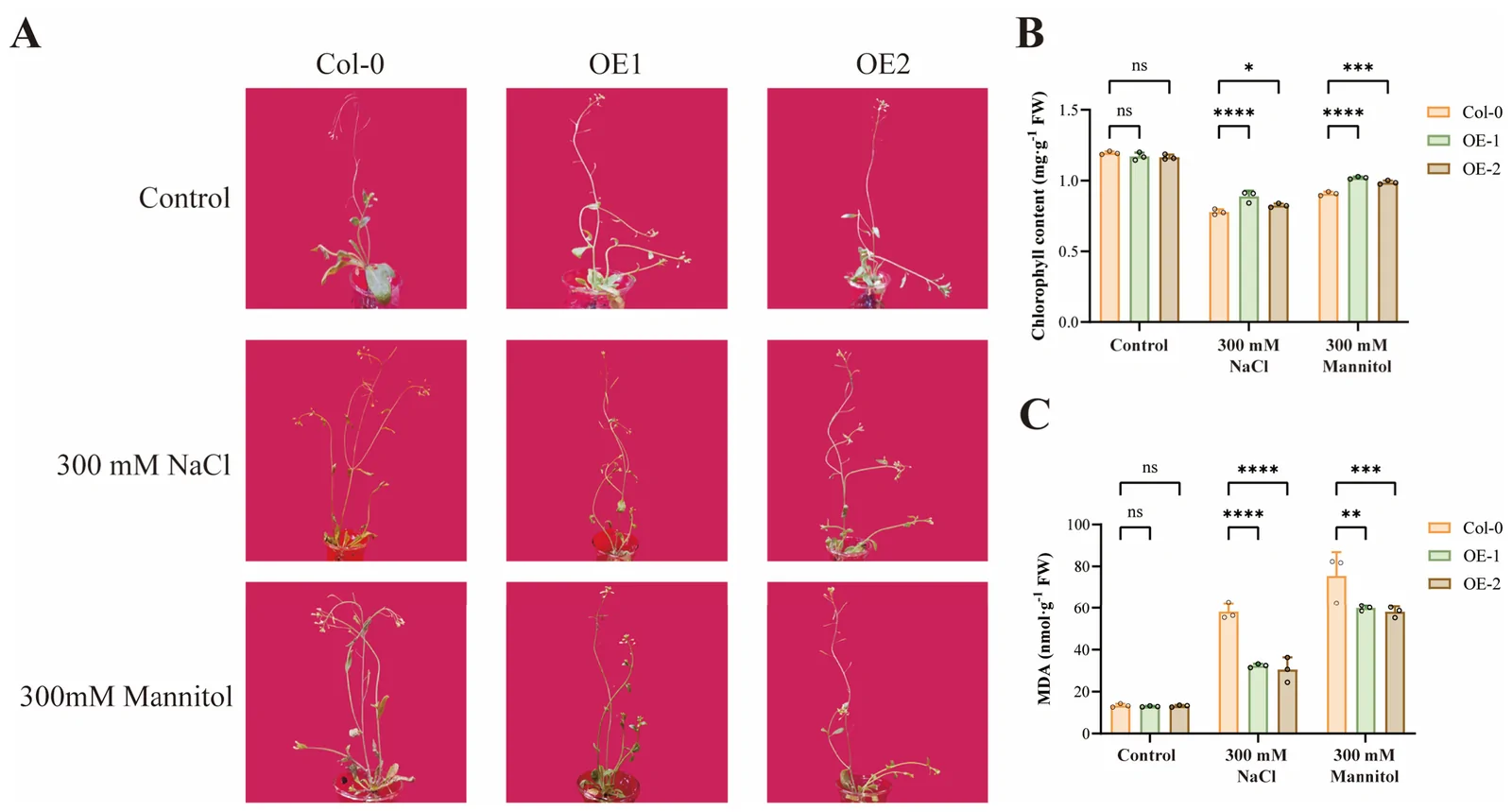
*SiLEA_2-6*-overexpressing *Arabidopsis* showed enhanced tolerance to salt and osmotic stress at the reproductive stage. (**A**) Representative phenotypes of 7-week-old bolting WT (Col-0), OE1, and OE2 plants (with initial flower buds) grown in nutrient solution under control conditions, 300 mM NaCl, or 300 mM mannitol for 48 h. (**B**) Chlorophyll content (mg·g^−1^·FW) under control, NaCl, and mannitol treatments. (**C**) MDA content (nmol·g^−1^·FW) under control, NaCl, and mannitol treatments. Data are presented as mean ± SD (*n* = 3). Statistical significance was determined using two-way ANOVA with Dunnett’s post-hoc test. ns, not significant; *, *p* < 0.05; **, *p* < 0.01; ***, *p* < 0.001; ****, *p* < 0.0001.

At the reproductive stage (7-week-old bolting plants with initial flower buds), WT (Col-0), OE1, and OE2 plants showed comparable phenotypes, chlorophyll contents, and MDA levels under control hydroponic conditions (Figure 6A–C). When exposed to 300 mM NaCl for 48 h, WT plants exhibited more severe wilting and loss of turgor than OE1 and OE2; accordingly, WT leaves had significantly lower chlorophyll content and markedly elevated MDA levels, whereas transgenic lines better retained greenness and membrane integrity than WT. Similarly, after 300 mM mannitol treatment for 48 h, WT plants displayed pronounced wilting and extensive browning, with significantly reduced chlorophyll content and increased MDA accumulation compared to OE1 and OE2. These results indicate that *SiLEA_2-6* overexpression sustains photosynthetic pigment retention and limits lipid peroxidation under salt and osmotic stress at the reproductive stage, extending the protective role of *SiLEA_2-6* beyond the vegetative stage.

### 2.7. SiLEA_2-6 Shows Stress-Altered Distribution Patterns in Arabidopsis Leaf Cells

**Figure 7:**
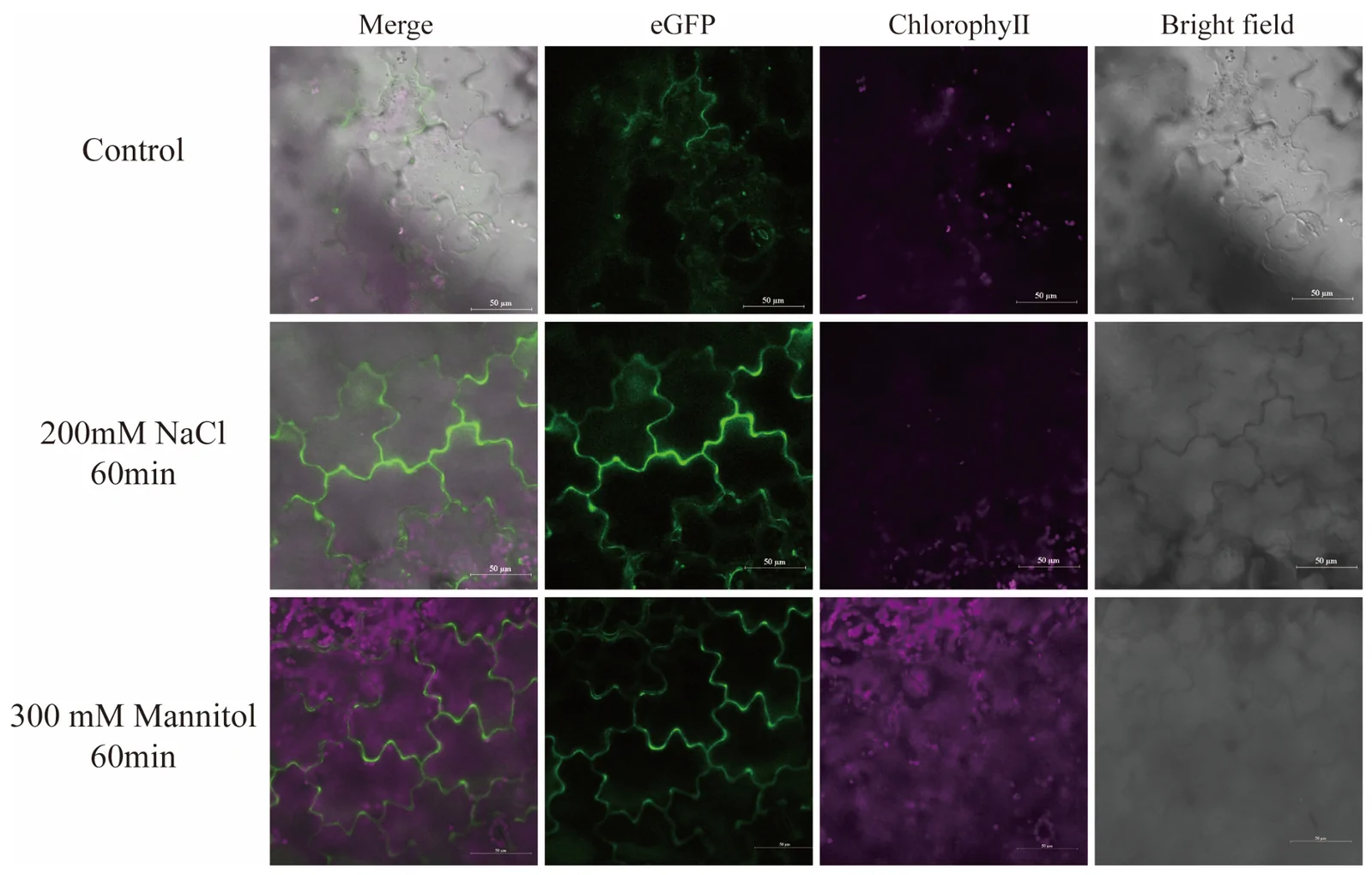
Subcellular localization of SiLEA_2-6 in *Arabidopsis* leaf epidermal cells under control and short-term stress conditions. Confocal images of leaf discs from three-week-old OE1 and OE2 plants showing merged, SiLEA_2-6 fluorescence (eGFP channel), chlorophyll autofluorescence, and bright-field channels under control, 200 mM NaCl, and 300 mM mannitol treatments for 60 min. Scale bars, 50 µm. Images were acquired using confocal microscopy with eGFP excitation at 488 nm and chlorophyll autofluorescence excitation at 640 nm.

To directly observe the subcellular distribution of SiLEA_2-6 under stress, leaf discs from three-week-old OE1 and OE2 plants were incubated in control solution, 200 mM NaCl, or 300 mM mannitol for 60 min before confocal imaging. Under control conditions, SiLEA_2-6 fluorescence displayed a diffuse cytoplasmic distribution with punctate structures throughout the cell and visible signal at the cell periphery (Figure 7). After 60 min of NaCl or mannitol treatment, the peripheral signal intensified markedly, producing brighter outlines of epidermal cell boundaries. Enhanced signal was also observed at guard cells and stomatal-adjacent regions. As a control, transient expression of free 35S-eGFP in *Arabidopsis* leaf epidermal cells under identical conditions showed uniform cytoplasmic and nuclear distribution, with no stress-responsive redistribution (Figure S1). This contrast indicates that the altered distribution pattern of SiLEA_2-6-eGFP is specific to the fusion protein and not an artifact of passive GFP diffusion. Because the fusion protein is driven by the constitutive 35S promoter, the changed distribution cannot be attributed to stress-induced transcriptional activation. Rather, it reflects a rapid stress-associated redistribution or increased peripheral association of pre-existing SiLEA_2-6. However, in the absence of organelle-specific markers and plasmolysis assays, we cannot definitively assign the peripheral signal to the plasma membrane versus stress-induced changes in cytoplasmic or endomembrane distribution. The observed peripheral enrichment occurs within 60 min, preceding the physiological protection observed after 48 h (Section 2.5), and is consistent with a potential membrane-associated protective function during the early phase of salt or osmotic challenges. We note that the present confocal analyses are qualitative and represent observational data; future studies incorporating quantitative signal intensity measurements and independent biological replicates will be necessary to validate these patterns.

## 3. Discussion

LEA proteins are widely recognized as IDPs that protect cells from dehydration and osmotic stress through multiple mechanisms, including membrane stabilization, ROS scavenging, and chaperone-like activity [10,24]. Among the eight LEA subfamilies, LEA_2 is distinguished by its high hydrophobicity and relatively low sequence conservation compared to other LEA groups [9,25]. Although several LEA_2 members have been shown to confer freezing or drought tolerance when heterologously expressed [15,19,20,21], their functional characterization under salt and osmotic stress, particularly at the protein subcellular dynamics level, remains limited. The present study provides evidence for the stress-inducible peripheral association of SiLEA_2-6, an LEA_2 protein, in a heterologous *Arabidopsis* system.

In the present study, overexpression of *SiLEA_2-6* in *Arabidopsis* Col-0 conferred enhanced tolerance to salt and osmotic stress across multiple developmental stages, from seed germination to the reproductive phase. At the germination stage, OE lines maintained higher germination rates under 150 mM NaCl and 250 mM mannitol, consistent with previous reports that LEA protein accumulation promotes seed vigor and stress resilience [26]. During vegetative growth, OE roots were approximately 0.4 cm longer than WT under 150 mM NaCl, and the difference increased under more severe stress conditions. This root protection correlates with the well-documented role of LEA_2 proteins in maintaining cell turgor and membrane integrity under water deficit conditions [23,27]. At the reproductive stage, 7-week-old OE plants retained chlorophyll and suppressed MDA accumulation under 300 mM NaCl and mannitol. These results demonstrate that SiLEA_2-6 can protect plants from salt and osmotic damage throughout their entire life cycle, extending beyond the seedling-stage focus of most previous LEA overexpression studies. Future studies should include Western blot analysis using anti-GFP antibodies to confirm SiLEA_2-6–eGFP protein accumulation and to assess whether protein levels correlate with the observed phenotypic variation among independent overexpression lines. Additionally, temporal expression profiling of *SiLEA_2-6* transcripts under different stress durations would provide insight into the dynamics of stress-responsive upregulation.

The physiological basis of SiLEA_2-6-mediated protection likely involves multiple mechanisms. First, the reduced REL and suppressed MDA accumulation in OE lines under drought conditions indicated that the transgenic plants maintained better plasma membrane integrity than WT. This is consistent with the proposed role of LEA_2 proteins in membrane protection, potentially through their interactions with membrane lipids [10,28]. Second, the elevated proline accumulation in OE plants under drought conditions suggests that SiLEA_2-6 may either facilitate osmotic adjustment or protect proline biosynthesis enzymes from stress-induced denaturation, consistent with the chaperone-like activity reported for other IDPs [8,11]. Third, chlorophyll retention in both detached leaf discs and reproductive-stage plants implies that SiLEA_2-6 preserves the photosynthetic apparatus from salt/osmotic damage, likely by limiting thylakoid membrane destabilization, a function previously attributed to LEA_2 members in pea mitochondria (LEAM) and plastid-localized LEA23/LEA24 [10,29].

A particularly striking observation in this study was the stress-inducible peripheral enrichment of *SiLEA_2-6*–eGFP, where the signal transitions from diffuse cytoplasmic distribution with intracellular punctate structures to increased peripheral association within 60 min of salt or osmotic treatment. This rapid alteration in distribution suggests that SiLEA_2-6 does not merely act as a constitutive stress buffer but can dynamically respond to osmotic cues, with notably enhanced signal at the guard cell periphery. Accumulating evidence supports the membrane-protective functions of LEA proteins under stress conditions. For example, some LEA family members can insert into membranes via amphipathic α-helices (e.g., LEAM, a group 3 LEA protein) [30,31,32], interact with lipid vesicles through specialized segments (e.g., K-segments in dehydrins/group 2 LEA) [33] or retain intrinsic structural disorder and adopt protective conformations under freeze–thaw cycles (e.g., PM1) [28]. Given these precedents, it is plausible—though not yet directly demonstrated—that SiLEA_2-6 may undergo a stress-responsive conformational change that exposes hydrophobic patches, enabling transient peripheral association and localized stabilization of cellular membranes. This may be particularly relevant at sites such as guard cells and stomatal-adjacent regions, where prominent peripheral signal enhancement was observed. This possibility is further supported by the fact that SiLEA_2-6 shares conserved LEA_2 domain features with AtLEA14 (Figure S2), a member of the family implicated in stress-responsive membrane processes [34]. However, whether SiLEA_2-6 actually undergoes conformational rearrangement, binds specific lipids, or utilizes particular domains for membrane association remains to be directly demonstrated. Definitive evidence for these mechanisms will require future experiments such as liposome-binding assays, circular dichroism spectroscopy, or plasmolysis-based subcellular localization with organelle markers.

In summary, SiLEA_2-6 from *S. involucrata* is a functional LEA_2 protein that enhances salt and osmotic tolerance in *Arabidopsis*. This protective effect may involve integrated physiological protection and stress-altered peripheral distribution of the protein. Our findings add to the growing evidence that LEA_2 proteins confer tolerance not only to cold/freezing but also to salt and osmotic stress, and suggest that stress-inducible peripheral association is a plausible, though not yet directly demonstrated, mechanism for LEA_2 members in a heterologous plant system. Future studies should address the molecular details of this peripheral redistribution, including whether specific lipid components or post-translational modifications mediate membrane targeting, and explore the potential of *SiLEA_2-6* as a genetic resource for improving crop resilience to salinity and drought stress.

## 4. Materials and Methods

### 4.1. Plant Materials and Growth Conditions

*Arabidopsis* ecotype Columbia-0 (Col-0) was used as the wild type. Seeds were surface-sterilized in 2 mL microcentrifuge tubes by sequential washing with sterile water, 75% (*v*/*v*) ethanol for 45 s, and 5% (*v*/*v*) sodium hypochlorite for 7 min, with sterile water rinses between each step. For in vitro assays, sterilized seeds were sown on 1/2 Murashige and Skoog (MS) solid medium, stratified at 4 °C in the dark for 2 d, then transferred to a growth chamber (16 h light/8 h dark, 22 ± 2 °C, 35–45% relative humidity, ~8000 lx). For soil cultivation, kanamycin-resistant seedlings grown on 1/2 MS medium were transplanted into soil and grown under the same photoperiod and temperature with 55–65% relative humidity.

### 4.2. Vector Construction and Agrobacterium-Mediated Transformation

The open reading frame (ORF) of *SiLEA_2-6* was identified using the NCBI ORF Finder tool (https://www.ncbi.nlm.nih.gov/orffinder/ (accessed on 3 May 2021)). Gene-specific primers were designed using Primer 5 software (Premier Biosoft, Palo Alto, CA, USA) to amplify the full-length coding sequence. *BamH* I and *Pst* I restriction sites were introduced at the 5′ and 3′ ends of the forward and reverse primers, respectively, for subsequent directional cloning. The primer sequences are presented in Table S1.

The amplified *SiLEA_2-6* fragment was ligated into the pMD19-T intermediate vector (Takara, Dalian, China), and the ligation product was transformed into *E. coli* DH5α competent cells. Positive clones were selected on ampicillin-containing (100 mg/L) medium and verified by colony PCR and Sanger sequencing to confirm the absence of mutations. The pCAMBIA2300-eGFP-OCS vector was a laboratory-modified binary vector based on pCAMBIA2300. The amplified *SiLEA_2-6* fragment was digested with *BamH* I and *Pst* I and ligated into the corresponding sites upstream of eGFP to generate the pCAMBIA2300-*SiLEA_2-6*-eGFP-OCS fusion construct. The ligation reaction (10 μL total volume: 1 μL T4 DNA ligase, 1 μL 10× ligation buffer, insert fragment and digested vector at a molar ratio of approximately 3:1, nuclease-free water to 10 μL) was incubated at 16 °C for 12–16 h. The verified construct was introduced into *Agrobacterium tumefaciens* strain GV3101 by freeze–thaw, and *Arabidopsis* Col-0 plants were transformed by the floral dip method. Transgenic seeds (T_1_) were selected on 1/2 MS medium containing kanamycin (50 mg/L). Resistant seedlings were transferred to soil and self-pollinated to obtain T_3_ lines for subsequent experiments.

### 4.3. Molecular Identification of Transgenic Lines

#### 4.3.1. Plant Genomic DNA Extraction and PCR Analysis

Genomic DNA was extracted from the leaf tissues of T_3_ transgenic lines and Col-0 using the Plant Genomic DNA Extraction Kit (Tiangen Biotech, Beijing, China) following the manufacturer’s protocol. PCR amplification was performed to detect the integration of the *SiLEA_2-6* coding sequence using gene-specific primers (Table S1). Each 20 µL reaction mixture contained 10 µL 2× PCR Mix (Vazyme Biotech, Nanjing, China), 0.5 µL forward primer (10 µM), 0.5 µL reverse primer (10 µM), 1 µL template DNA, and 8 µL ddH_2_O. The PCR program was as follows: initial denaturation at 94 °C for 4 min; 30 cycles of 94 °C for 30 s, 58 °C for 30 s, and 72 °C for 45 s; final extension at 72 °C for 7 min; held at 4 °C. PCR products were separated by 1.0% agarose gel electrophoresis and visualized under UV light after staining. Col-0 and a no-template control were included as negative controls.

#### 4.3.2. RNA Extraction, cDNA Synthesis, and RT-PCR Analysis

Total RNA was extracted from leaf tissues using the Plant Total RNA Extraction Kit (Tiangen Biotech, Beijing, China) according to the manufacturer’s instructions. RNA concentration and purity were determined with a NanoDrop 2000 spectrophotometer (Thermo Fisher Scientific, Waltham, MA, USA). Genomic DNA contamination was removed by treatment with RNase-free DNase I. First-strand cDNA was synthesized from total RNA using M-MLV reverse transcriptase (Takara, Dalian, China) with oligo(dT) primers. PCR was performed to amplify the *SiLEA_2-6*–eGFP fusion transcript and the *SiLEA_2-6* transcript using corresponding primer pairs (Table S1). The PCR mixture and cycling conditions were the same as described in Section 4.3.1. PCR products were analyzed by 1.0% agarose gel electrophoresis. For qRT-PCR validation, relative expression of SiLEA_2-6 was quantified using SYBR Green-based real-time PCR on a Bio-Rad. *Arabidopsis Actin2* (*AtACT2*) served as the internal reference. Col-0 was used as a negative control to confirm the absence of endogenous *SiLEA_2-6* expression in wild-type *Arabidopsis*.

### 4.4. Seed Germination and Root Growth Assays

To evaluate the response of *SiLEA_2-6* overexpressing lines to salt and osmotic stress at the seed germination stage, WT (Col-0) and OE1/OE2 T_3_ seeds were surface-sterilized as described in Section 4.1 and sown on 1/2 MS solid medium supplemented with NaCl (0, 100, or 150 mM) or mannitol (0, 150, or 250 mM). Seeds were individually spotted onto plates using sterile, heat-dried toothpicks to ensure uniform sowing. After 2 days of stratification at 4 °C in the dark, plates were transferred to a growth chamber (Section 4.1). Germination rate was scored on day 6 post-stratification as the percentage of seeds with visible radicle emergence. Root length was measured on day 8 from digital images using ImageJ (version 1.54f, National Institutes of Health, Bethesda, MD, USA, https://imagej.nih.gov/ij/, accessed on 22 August 2023). Each treatment included 3 biological replicates with 20 seeds per replicate.

### 4.5. Whole-Plant Drought and Salt Stress Treatments

Three-week-old WT (Col-0) and OE1/OE2 plants grown in soil were subjected to drought and salt stress treatments. For salt stress, plants were irrigated with 300 mM NaCl solution at 50 mL per pot (7-cm square pot) every 2 days for 15 days, with well-watered plants receiving an equal volume of water serving as the control. Phenotypes were photographed on day 15. For drought stress, irrigation was withheld for 15 days to simulate drought conditions, while control plants were watered normally. After 15 days of drought, plants were rewatered and allowed to recover for 5 days, at which point recovery phenotypes were photographed. Physiological parameters (REL, chlorophyll content, MDA content, and proline content) were measured from leaf samples collected on day 15 of treatment, as described in Section 4.6.

### 4.6. Physiological Measurements

#### 4.6.1. Relative Electrolyte Leakage (REL)

Fresh leaf samples (0.1 g) were cut into small pieces and immersed in 10 mL ultrapure water in a 20 mL tube. Tubes were sealed with parafilm and incubated at room temperature for 24 h. The conductivity of the solution (R1) was measured using an EC215 conductivity meter (HANNA Instruments, Woonsocket, RI, USA). Tubes were then boiled for 1 h until conductivity stabilized, and the final conductivity (R2) was measured. REL was calculated as: REL (%) = R1/R2 × 100%.

#### 4.6.2. Chlorophyll Content

Leaf samples (0.1 g) were collected from the same positions before and after treatment and extracted in 10 mL 95% ethanol in the dark overnight until the tissues were completely decolorized. The absorbance of the extract was measured at 665 nm and 649 nm using a 721E visible spectrophotometer (Shanghai Spectrum Instruments Co., Ltd., Shanghai, China). Chlorophyll content (mg·g^−1^ FW) was calculated as follows:

$$\mathrm{Chlorophy}\;\mathrm{II}\;\mathrm{content}=\frac{(6.63\times A_{665}+18.08\times A_{649})\times V}{\left(W\times 10^{3}\right)}$$

where *V* is the extraction volume (mL), and *W* is the fresh weight (g).

#### 4.6.3. Malondialdehyde (MDA) Content

Fresh leaf tissue (0.1 g) was homogenized in 2 mL of 5% trichloroacetic acid (TCA), and the homogenate was brought to 10 mL with additional 5% TCA. After centrifugation at 5000 rpm for 10 min, 2 mL of the supernatant was mixed with 2 mL of 0.67% thiobarbituric acid (TBA). The mixture was heated in a boiling water bath for 20 min, cooled to room temperature, and centrifuged at 5000 rpm for 10 min. Absorbance was measured at 532 nm, 600 nm, and 450 nm. The MDA concentration (nmol·g^−1^ FW) was calculated as follows:

*MDA* = (6.45 × (*A*_532_ − *A*_600_) − 0.56 × *A*_450_) × *V*/*W*

where *V* and *W* are as defined in Section 4.6.2.

#### 4.6.4. Proline Content

Fresh leaf tissue (0.1 g) was homogenized in 2 mL of 80% ethanol, and the homogenate was adjusted to 10 mL with additional 80% ethanol. After incubation at 80 °C for 20 min, the mixture was centrifuged at 5000 rpm for 10 min to separate the supernatant. A 2 mL aliquot of the supernatant was mixed with 2 mL of glacial acetic acid and 2 mL of 2.5% acidic ninhydrin, followed by heating in a boiling water bath for 15 min. After cooling to room temperature, the absorbance was measured at 520 nm. The proline concentration was determined with reference to a standard curve (0–20 µg proline; Table S2).

### 4.7. Detached Leaf Disc Assays

To further assess the response of *SiLEA_2-6* to salt and osmotic stress at the vegetative stage, leaf discs were prepared from healthy three-week-old WT (Col-0) and OE1/OE2 plants. Leaf discs (6 mm in diameter) were punched from uniform positions on the leaves using a cork borer. The discs were incubated in solutions containing NaCl (0, 200, or 300 mM) or mannitol (0 or 300 mM). After 48 h of treatment, phenotypic changes were photographed, and chlorophyll content was measured as described in Section 4.6.2.

### 4.8. Reproductive-Stage Whole-Plant Stress Assay

Seven-week-old WT (Col-0) and OE1/OE2 plants with visible floral buds were carefully uprooted from vermiculite–coconut coir substrate, and the roots were gently washed with tap water to remove residual medium. Plants of similar size and growth status were selected for uniformity. Plants were pre-equilibrated in 1/2 MS liquid medium for 24 h to recover from root disturbance, then transferred to the same medium supplemented with 300 mM NaCl or 300 mM mannitol. Phenotypic differences were evident within 24 h. After 48 h of treatment, phenotypes were photographed, and physiological parameters (chlorophyll content and MDA content) were measured as described in Section 4.6.

### 4.9. Confocal Microscopy

Leaf discs (6 mm in diameter) were punched from three-week-old OE1 and OE2 plants and incubated in 200 mM NaCl or 300 mM mannitol solution for 60 min. As a control, wild-type Col-0 plants were transiently infiltrated with *Agrobacterium* carrying the 35S-eGFP construct. After 48 h, leaf discs were excised and incubated in the same solutions for 60 min prior to confocal microscopy. After treatment, leaf discs were mounted on slides and observed under a laser confocal microscope (Nikon AXR, Nikon Corporation, Tokyo, Japan) with a 40× objective. eGFP fluorescence was excited at 488 nm and detected at 500–550 nm. Chlorophyll autofluorescence was excited at 640 nm and detected at 650–700 nm. Scale bars = 50 µm. Images were processed with NIS-Elements (Nikon Corporation, Tokyo, Japan).

### 4.10. Statistical Analysis

All experiments were performed in at least three biological replicates. Data are presented as the mean ± SD. Relative expression data were analyzed using one-way analysis of variance (one-way ANOVA) followed by Tukey’s multiple comparison test to determine significant differences among genotypes. All other phenotypic and physiological data were analyzed by two-way ANOVA followed by Dunnett’s post-hoc test. Differences were considered significant at *p* < 0.05. Statistical analyses were performed using GraphPad Prism version 8.0 (GraphPad Software, San Diego, CA, USA).

## 5. Conclusions

In this study, we cloned and characterized *SiLEA_2-6*, a LEA_2 (PF03168) gene, from *S. involucrata*, and demonstrated its function in enhancing salt and osmotic stress tolerance in transgenic *Arabidopsis*. The main conclusions are as follows:

Heterologous overexpression of *SiLEA_2-6* in *Arabidopsis* enhances tolerance to salt and osmotic stress, as evidenced by improved germination rates, root growth, and survival percentage under stress conditions. *SiLEA_2-6* protein localizes to the cytoplasm and intracellular punctate structures, with stress-inducible peripheral signal enhancement. Under salt or osmotic stress, the fusion protein exhibits stress-inducible enrichment at the cell periphery within 60 min, suggesting a potential membrane-associated protective function. Physiological analyses indicate that *SiLEA_2-6* overexpression reduces ion leakage and MDA accumulation while enhancing relative water content and chlorophyll retention, supporting its protective function against cellular damage. These findings identify SiLEA_2-6 as a stress-associated LEA protein with stress-responsive subcellular distribution and demonstrate its potential for improving abiotic stress tolerance in plants.

## Data Availability

The *SiLEA_2-6* sequence has been assigned GenBank accession PZ794267, scheduled for public release on 1 December 2026 (currently under NCBI hold). All other data supporting the findings of this study are included in the article and its Supplementary Materials.

## Supplementary Materials

The following supporting information can be downloaded at: https://www.mdpi.com/article/10.3390/plants15203154/s1, Table S1. Primer sequences used for SiLEA_2-6 cloning and vector construction. Table S2. Standard curve for proline assay. Figure S1. Subcellular distribution of free 35S-eGFP in Arabidopsis leaf discs under control and stress conditions. Confocal images of leaf discs from three-week-old wild-type (Col-0) plants transiently expressing free 35S-eGFP, incubated in control solution, 200 mM NaCl, or 300 mM mannitol for 60 min. Panels show merged images, eGFP fluorescence (green), chlorophyll autofluorescence (red), and bright-field channels (left to right). Free eGFP exhibits uniform cytoplasmic and nuclear distribution under all conditions, with no stress-induced peripheral enrichment or redistribution. Scale bars: 50 μm. Images were acquired by confocal microscopy with eGFP excitation at 488 nm and chlorophyll autofluorescence excitation at 640 nm. Note: In some cells, free eGFP exhibited a punctate appearance due to (i) chlorophyll autofluorescence bleed-through at high detector gain—regions with strong red autofluorescence were avoided during analysis; (ii) overexpression-induced cytoplasmic aggregation under the strong 35S promoter; and (iii) mild cellular stress from protoplast isolation/imaging. All images within this figure were acquired using strictly identical microscope settings (laser power, detector gain, exposure time) for control, NaCl-treated, and mannitol-treated samples. Control leaf discs were sampled from regions closer to the infiltration site, which may have experienced greater mechanical impact, potentially explaining the more pronounced punctate pattern in control images. While direct quantitative comparison with main experimental figures is not appropriate due to separate imaging sessions, relative patterns within this figure are internally consistent. We acknowledge that co-localization with organelle-specific markers was not performed; this limitation is discussed in the main text. Figure S2. Domain architecture of SiLEA_2-6 predicted by InterProScan. The protein contains a LEA14 superfamily domain (InterPro: IPR045043) and a LEA_2 domain (InterPro: IPR004864; Pfam: PF03168), confirming its classification within the LEA_2 subfamily and its relationship to AtLEA14 (At1g01470), which belongs to the same LEA14 superfamily. qRT-PCR.
