# Supplementary material for: Overexpression of SiLEA_2-6 from Saussurea involucrata Enhances Salt and Osmotic Tolerance in Arabidopsis thaliana
Source: Plants (Basel). 2026 Oct 15;15(20):3154. doi: 10.3390/plants15203154 (PMC13709042; doi:10.3390/plants15203154)
Supplement: Supplementary file 1 [file plants-15-03154-s001.zip › Supplementary Tables and Figures.pdf]

Table S1. Primer sequences used for *SiLEA_2-6* cloning and vector construction.

Primer ID	Sequence (5' – 3')
SiLEA_2-6-F	GGATCCATGTCAATGCACGCAAAGAC
SiLEA_2-6-R	CTGCAGCTCAACGGTGCAAGAGTTC
SiLEA_2-6-eGFP-R	CTGCATTACTTGTACAGCTCGTCCATG
qRT-PCR-SiLEA_2-6-F	TGAACTGGAGCAGCGAGAAC
qRT-PCR-SiLEA_2-6-R	TGGTGGGTTTGTAGACGATGG

Note: Restriction sites are underlined: *BamH* I (GGATCC) in SiLEA_2-6-F; *Pst* I (CTGCAG) in SiLEA_2-6-R. The native stop codon of *SiLEA_2-6* was omitted in the reverse primer (SiLEA_2-6-R) to enable in-frame fusion with eGFP. SiLEA_2-6-eGFP-R was designed to verify the integration of the SiLEA_2-6–eGFP fusion fragment into the transgenic lines.

Table S2. Standard curve for proline assay.

Reagents	Tube0	Tube 1	Tube 2	Tube 3	Tube 4	Tube 5	Tube 6
Proline standard solution (mL)	0	0.2	0.4	0.6	0.8	1.0	2.0
Distilled water (mL)	2	1.8	1.6	1.4	1.2	1.0	0
Glacial acetic acid (mL)	2	2	2	2	2	2	2
2.5% Acidic ninhydrin (mL)	2	2	2	2	2	2	2
Proline content (μg)	0	2	4	6	8	10	20

Note: All tubes were heated in a boiling water bath for 15 min. The absorbance was measured at 520 nm. The standard curve was plotted with proline content (μg) on the x-axis and absorbance (OD_{520}) on the y-axis.

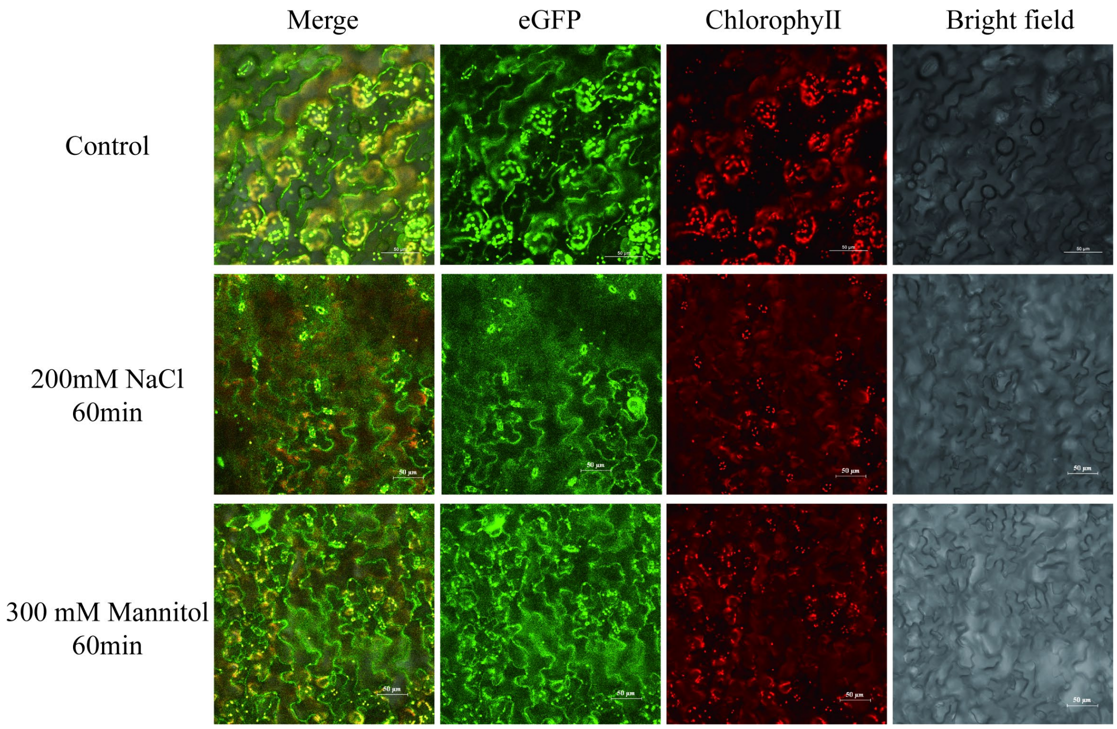

Figure S1. Subcellular distribution of free 35S-eGFP in Arabidopsis leaf discs under control and stress conditions. Confocal images of leaf discs from three-week-old wild-type (Col-0) plants transiently expressing free 35S-eGFP, incubated in control solution, 200 mM NaCl, or 300 mM mannitol for 60 min. Panels show merged images, eGFP fluorescence (green), chlorophyll autofluorescence (red), and bright-field channels (left to right). Free eGFP exhibits uniform cytoplasmic and nuclear distribution under all conditions, with no stress-induced peripheral enrichment or redistribution. Scale bars: 50 μm. Images were acquired by confocal microscopy with eGFP excitation at 488 nm and chlorophyll autofluorescence excitation at 640 nm. Note: In some cells, free eGFP exhibited a punctate appearance due to (i) chlorophyll autofluorescence bleed-through at high detector gain—regions with strong red autofluorescence were avoided during analysis; (ii) overexpression-induced cytoplasmic aggregation under the strong 35S promoter; and (iii) mild cellular stress from protoplast isolation/imaging. All images within this figure were acquired using strictly identical microscope settings (laser power, detector gain, exposure time) for control, NaCl-treated, and mannitol-treated samples. Control leaf discs were sampled from regions closer to the infiltration site, which may have experienced greater mechanical impact, potentially explaining the more pronounced punctate pattern in control images. While direct quantitative comparison with main experimental figures is not appropriate due to separate imaging sessions, relative patterns within this figure are internally consistent. We acknowledge that co-localization with organelle-specific markers was not performed; this limitation is discussed in the main text.

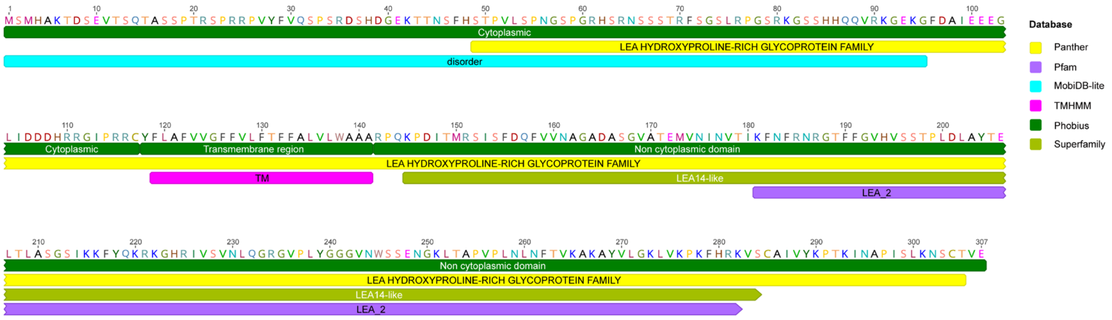

Figure S2. Domain architecture of SiLEA_2-6 predicted by InterProScan. The protein contains a LEA14 superfamily domain (InterPro: IPR045043) and a LEA_2 domain (InterPro: IPR004864; Pfam: PF03168), confirming its classification within the LEA_2 subfamily and its relationship to AtLEA14 (At1g01470), which belongs to the same LEA14 superfamily.
